# Transition of inflammatory bowel disease patients from pediatric to adult care: an observational study on a joint-visits approach

**DOI:** 10.1186/s13052-021-00977-x

**Published:** 2021-01-28

**Authors:** Antonio Corsello, Daniela Pugliese, Fiammetta Bracci, Daniela Knafelz, Bronislava Papadatou, Marina Aloi, Salvatore Cucchiara, Luisa Guidi, Antonio Gasbarrini, Alessandro Armuzzi

**Affiliations:** 1 Fondazione Policlinico Universitario A. Gemelli IRCCS, OU Internal Medicine and Gastroenterology, Largo A. Gemelli, 00168 Rome, Italy; 2 Hepatology, Gastroenterology and Nutrition Unit, Bambino Gesù Children’s Hospital, Rome, Italy; 3 Pediatric Gastroenterology and Hepatology Unit, Sapienza University, Rome, Italy; 4 Università Cattolica del Sacro Cuore, Rome, Italy

**Keywords:** Transition, Pediatric IBD, Crohn’s disease, Ulcerative colitis, TRAQ, SIBDQ

## Abstract

**Background:**

Transition from pediatric to adult care of patients affected by Inflammatory Bowel Disease (IBD) is a critical step that needs specific care and multidisciplinary involvement. The aim of our study was to evaluate the outcome of the transition process of a cohort of IBD patients, exploring their readiness and the possible impact on quality of life.

**Methods:**

This observational study followed transitioned IBD patients from pediatric to adult care. Transition was carried-out through combined visits, jointly performed by the pediatrician and the adult gastroenterologist. Clinical data were collected before and after transition. A subgroup of patients was submitted to an anonymous online questionnaire of 38 items based on the validated questionnaires TRAQ and SIBDQ within the first 6 months from the beginning of the transition process.

**Results:**

Eighty-two patients with IBD were enrolled, with a mean age at transition of 20.2±2.7 years. Before transition, 40.2% of patients already had major surgery and 64.6% started biologics. At transition, 24% of patients were in moderate to severe active phase of their disease and 40% of them had already been treated with ≥ 2 biologics. The mean score of the TRAQ questionnaires collected is 3.4±1.5 and the mean score of SIBDQ is 53.9±9.8. A significant association was found between a TRAQ mean score > 3 and a SIBDQ > 50 (*p*=0.0129). Overall, 75% of patients had a positive opinion of the transition model adopted.

**Conclusions:**

A strong association has been found between TRAQ and SIBDQ questionnaires, showing how transition readiness has a direct impact on the quality of life of the young adult with IBD.

## Introduction

Up to the 25% of patients affected by Inflammatory Bowel Disease (IBD) receive their diagnosis before the age of 20 years, with a constantly growing incidence of the diagnosis made during childhood [1]. Pediatric IBD shows peculiar features compared to adult one, including a more extensive disease and a more frequent upper gastrointestinal tract involvement. Moreover, patients with an onset diagnosed during childhood are recently more likely to receive immunosuppressive or biological therapies since from the moment of diagnosis [2]. Children with IBD are found to have frequent mood disorders and are considered at higher risk for difficulties in social, family and school functioning [3]. A crucial phase in the management of children with IBD is the transition from the pediatric to the adult IBD care system, requiring a full achievement of maturity and self-management skills, which not occur at the same age for each individual [4]. The “ideal” patient for a transition process should be a young independent adult in a clinical remission phase, with no change of medical therapy or surgical intervention necessarily planned [5].

The guideline of the British Society of Gastroenterology on transition of young adults with chronic digestive disease recommends a shared management between pediatrician and adult center, through an educational and conscious process that involves both the patient and his/her family, verifying the readiness of the young and his/her level of awareness [6]. The Transition Readiness Assessment Questionnaire (TRAQ) is one of the most useful tool (not disease specific) for assessing patient’s readiness to fulfill the transition from the pediatric health service to the adult care [7], exploring both the self-management domain (e.g. handling medications, arranging medical follow-up visits, managing finances, health insurance) and the self-advocacy one (e.g. communication with providers and managing activities of daily living and use of school and community resources). As far as IBD are concerned, age seems to be the best predictor of TRAQ score, and lower scores on the medication management section are associated with higher risk of nonadherence [8].

The Short Inflammatory Bowel Disease Questionnaire (SIBDQ) is a validated health-related quality of life (HRQoL) tool, designed to find out IBD symptoms, emotional status and limitation in social activities due to IBD symptoms in the last 2 weeks [9].

The aim of this study is to evaluate the outcomes of the transition process of consecutive patients transferred from two pediatric referral hospitals to an IBD adult hospital unit, in order to explore patients’ readiness and its association to the quality of life.

## Materials and methods

### Data collection

The study was carried out at IBD Unit - Fondazione Policlinico Universitario A. Gemelli IRCCS, Rome Italy. Consecutive patients with a previous diagnosis of pediatric IBD made within the age of 19, who transited from two different pediatric centers in Rome (Italy), the “Bambino Gesù Pediatric Hospital” (OPBG) and the “Policlinico Umberto I” on a period going from January 2014 to June 2019, were included. For each patient transition have been carried out through two joint visits, with the presence of both pediatric and adult gastroenterologist specialists.

The first visit occurred at the pediatric center, where patients, parents and doctors examined the previous medical history and planned the timing of transition, according to the patient’s clinical status and needs, evaluating his grade of maturity and awareness of disease.

The second visit occurred instead at the adult center, giving to the patients the possibility to discuss with doctors about future plans and therapies in a more autonomous and conscious way, weather in presence or not of parents according to their will. From that moment, follow-up of patients has been made by the adult gastroenterologist, with a maximum distance of 2 months within two different visits and with an average length of the follow-up period of 18 months before the end of the data collection.

Clinical data that were collected at the moment of enrolment have been gender, age at diagnosis and at time of transition, type and location of IBD according to the Montreal classification [10], previous surgery, previous and current therapies at time of transition, clinical disease activity at diagnosis and at transition visit (measured with Partial Mayo Score (PMS) for Ulcerative Colitis (UC) and with Harvey-Bradshaw Index (HBI) for Crohn’s Disease (CD) [11, 12]) and available endoscopic and imaging reports. Relapse was defined as worsening of symptoms, change of treatment or need for surgery.

Patients were then submitted to an anonymous online questionnaire of 38 items based on the validated questionnaires TRAQ and SIBDQ and administered within the first 6 months from the beginning of the transition process. Questions from TRAQ about health insurance covers had been removed, due to the differences of the Italian public healthcare system. Poor transition readiness has been defined as a TRAQ mean score ≤ 3 points out of 5. The SIBDQ consists instead of 10 questions, scored of a seven-point scale with higher scores indicating a better quality of life. Good HRQoL was defined as a score above 50 points out of 70 [13].

Two final questions were added in order to evaluate the quality of transition process: 1) When do you think the transition process from your pediatrician to your gastroenterologist should have taken place? 2) What do you think are the most important necessary conditions for an ideal transition?

The study protocol conforms to the ethical guidelines and it has been approved by the ethical committee of the centers involved. An informed consent has been obtained from all patients.

### Statistical analysis

Categorical variables were synthesized with frequencies and percentages, continuous variables with averages and the measurement of the standard deviation (SD). The statistical association of the events, the graphical representation of the same ones and the evaluation of the significance had been carried out with MATLAB and Statistics Toolbox (The MathWorks, Natick, Massachusetts, US). Associations with a *p*-value < 0.05 were considered significantly different from zero.

## Results

### Patients’ characteristics

#### Before transition

A total of 82 patients were enrolled, of which 57 came from the OPBG and 25 from the Policlinico Umberto I. Forty-two patients were males (51.2%) and 49 (59.8%) were affected by CD. The mean age at diagnosis was 11.8±3.5 years. Among 49 patients with CD, 32 patients (65.3%) had a penetrating or stricturing disease, 21 (42.9%) showed growth failure at the time of diagnosis and 15 (30.6%) had a perianal disease. Of 33 patients with UC, 26 (78.8%) had pancolitis.

Table 1 summarizes baseline patients’ characteristics.

**Table 1:** Baseline patients’ characteristics

**Number of Patients**	82
CD	49 (59.8%)
UC	33 (40.2%)
**Sex**	42 M, 40 F
CD	30 M (61.2%)
UC	21 F (63.6%)
**Age**	
At diagnosis	11.8±3.5 years
At transition	20.1±2.7 years
**Localization CD**	
Ileal- n (%)	9 (18%)
Colic- n(%)	6 (12%)
Ileo-colic- n(%)	30 (61%)
Upper GI- n (%)	4 (8%) exclusive 8 (16%) in addition
**Perianal Disease**	15 (30.6%)
**Phenotype Pattern CD**	
Inflammatory (B1)	17 (34.7%)
Stricturing (B2)	24 (49%)
Penetrating (B3)	8 (16.3%)
**Growth Failure**	21 (42.9%)
**Localization UC**	
Ulcerative Proctitis (E1)	1 (3%)
Distal colitis (E2)	6 (18.2%)
Pancolitis (E3)	26 (78.8%)

With regard to the medications taken before transition, 75% of patients received corticosteroids for more than 3 months during childhood. Fifty-six patients (68.3%) began an immunosuppressive therapy with thiopurines (still ongoing at transition only in 8 patients, 14.3%), and 9 patients received other types of immunosuppressants (cyclosporine, methotrexate, thalidomide).

Fifty-three patients (64.6%) started at least one biological therapy in pediatric age (that is ongoing in 62% of patients at time of transition) and 15 (28%) already tried ≥ 2 different types of biologics.

**Fig. 1:**
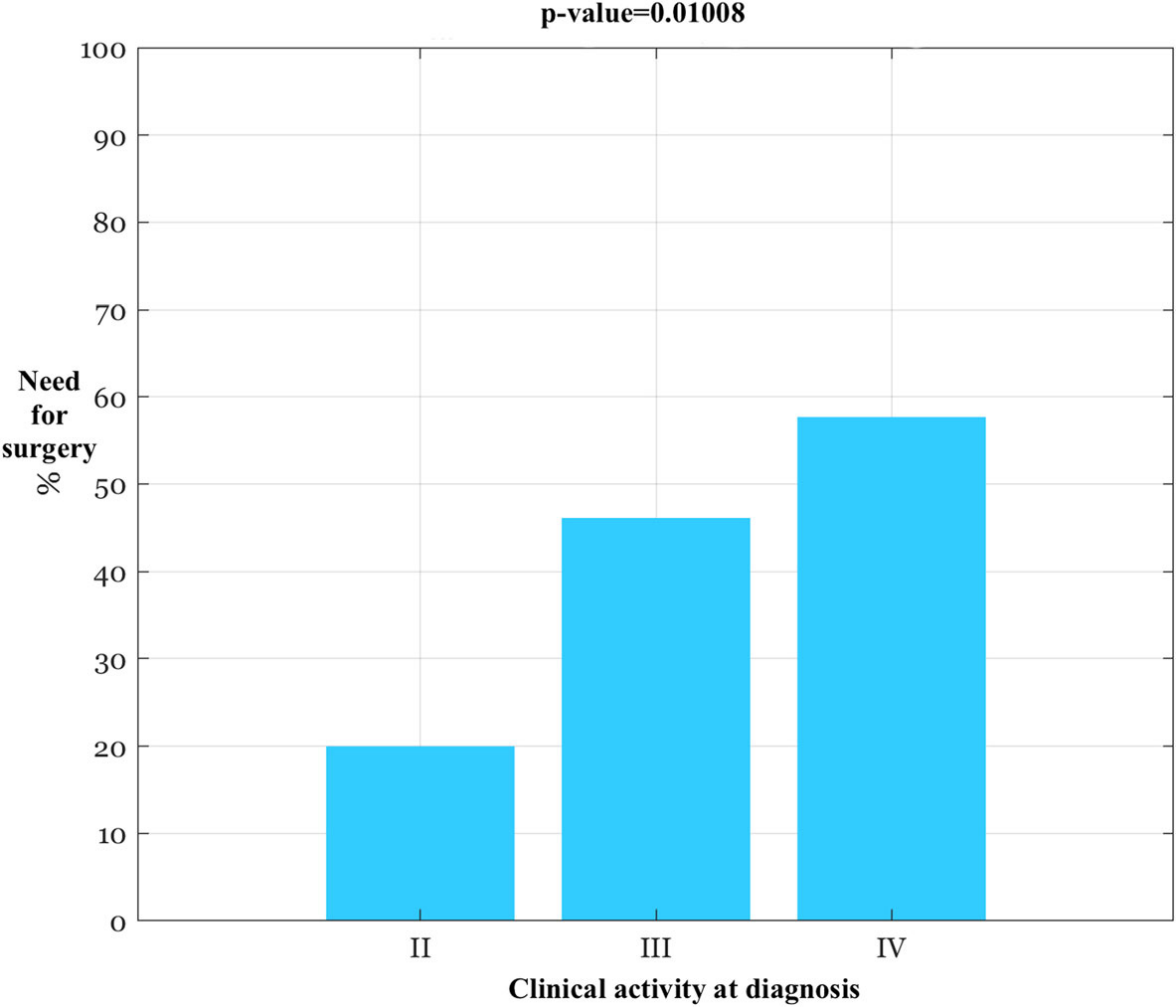
Association between probability of undergoing surgery before transition and clinical activity at diagnosis (HBI and PMS)

Up to the 40.2% of patients underwent one or more surgical intervention, more frequently for CD patients compared to UC (58% vs 15%, *P*=0.0003). Factor that associated with a higher risk of major surgeries occurred during the pediatric age have been a complicated (both stricturing and penetrating) CD (*p*=0.015), two or more types of biologics employed before transition (*p*=0.005) and a more severe clinical activity at diagnosis (*p*=0.001) (Fig. 1).

No significant difference (*p*> 0.05) has been found between patients coming from the two different centers in terms of mean age at diagnosis and transition, number and type of therapies and disease activity.

#### At time of transition

**Fig. 2:**
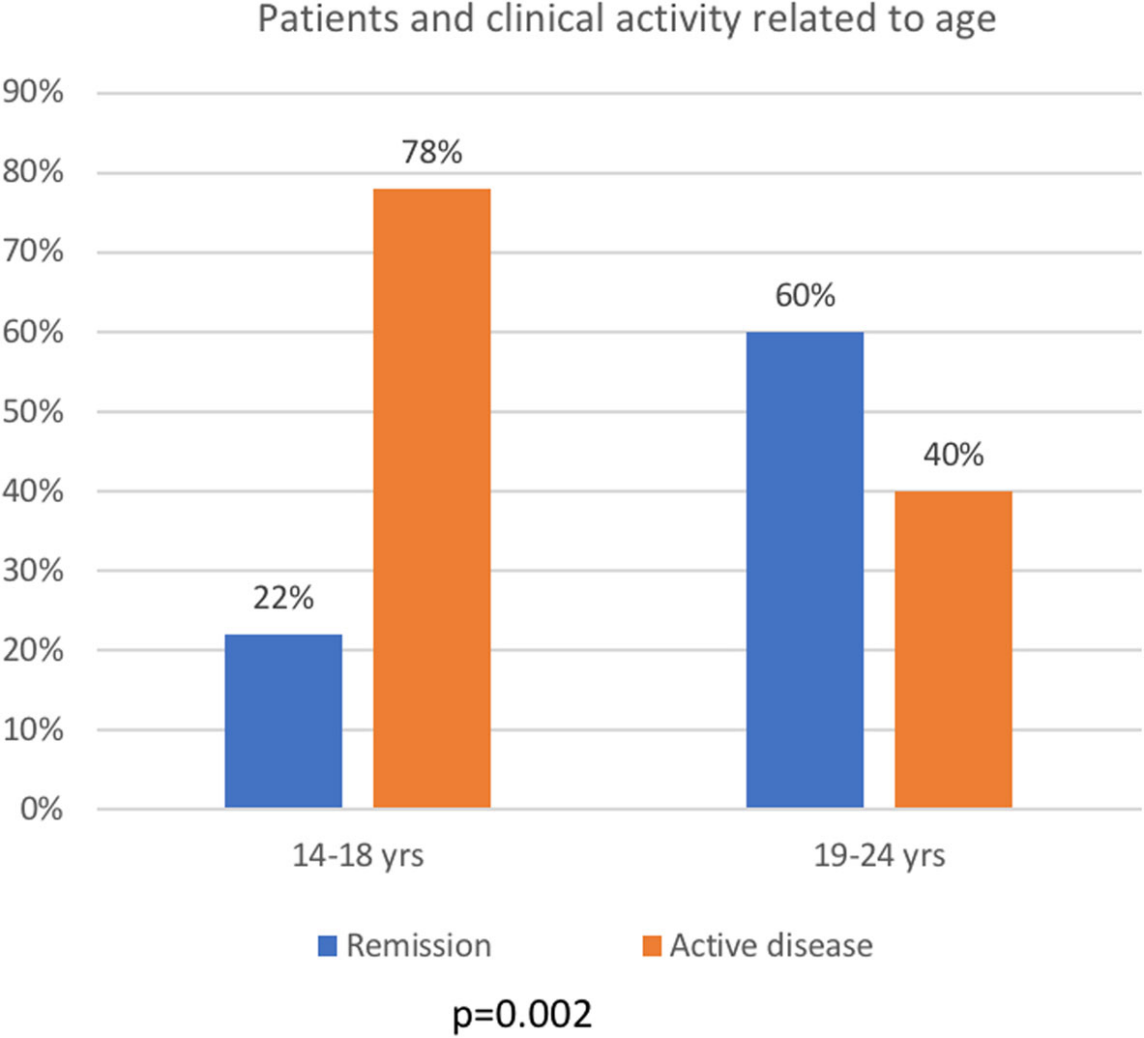
Transitioned patients divided by age and clinical disease activity

The average age observed at transition was 20.1±2.7 years, with an average time between the diagnosis and the beginning of transition to the adult center of 8.3±4.6 years. Only 22% of patients who carried out transition between ages 14 and 18 years were in a complete clinical remission phase, compared to the 60% of those who carried it out between ages 19 and 24 years (*p*=0.002, Fig. 2). No significant association was found between the age at transition and other parameters such as type of disease (*p*=0.89), previous surgery (*p*=0.44), clinical activity at diagnosis (*p*=0.78) and early onset (< 6 years) disease s (*p*=0.66).

Overall, 24% of patients carried out transition while on a moderate or severe disease activity of which 40% had already been treated with ≥ 2 biologics. 47.5% of patients had to change or start a new biologic therapy within 18 months after transition, and 21% of patients needed IBD-related surgery within the first 2 years after transition.

Transition while on moderate to severe active disease was significantly associated with a worsening of symptoms during the first year of follow-up visits at the adult center (p=0.002).

On the other hand, even if the 76% of patients carried out transition in a clinical remission phase or mild activity of disease (HBI or PMS = I or II), just the 80% of them really confirmed their clinical well-being with a complete endoscopic remission, and the 7.3% of their endoscopies revealed a moderate to severe endoscopic activity. Conversely, a complete endoscopic remission has been found to be related to a clinical remission with a correspondence of the 96%.

Furthermore, 64% of total patients carried out transition on a both clinical and endoscopic remission, and still maintain this condition at follow-up visits.

### Assessment patients’ readiness and quality of life

A selected group of fifty-three of patients, comparable for age and disease’s characteristics to the population of the study, answered the online anonymous questionnaires. Among them, up to 70% still go to follow-up visits at the adult center along with their parents, and 89% of them report that parents still have an important role in the management of therapy.

Table 2 summarizes patients’ answers.

**Table 2:** Transition Readiness Assessment Questionnaire (TRAQ) answers

Questions	Answers’ Mean Value (out of 5)
1. Do you know what to do if you are having a bad reaction to your medications	2.5 ± 1.2
2. Do you take medications correctly and on your own?	4,1 ± 1.1
3. Do you reorder medications before they run out?	4.6 ± 0.8
4. Do you call the doctor’s office to make an appointment?	3.2 ± 1.5
5. Do you follow-up on any referral for tests, check-ups or labs?	4 ± 1.2
6. Do you arrange for your ride to medical appointments?	2.2 ± 1.6
7. Do you call the doctor about unusual changes in your health (For example: Allergic reactions)?	3.9 ± 1.3
8. Do you know what your health insurance covers?	3.3 ± 1.1
9. Do you manage your money & budget household expenses (For example: use checking/debit card)?	2.7 ± 1.4
10. Do you fill out the medical history form, including a list of your allergies?	4.5 ± 0.7
11. Do you keep a calendar or list of medical and other appointments?	1.8 ± 1.2
12. Do you make a list of questions before the doctor’s visit?	1.9 ± 0.7
13. Do you tell the doctor or nurse what you are feeling?	4.1 ± 1.2
14. Do you answer questions that are asked by the doctor, nurse, or clinic staff?	4.8 ± 0.7
15. Do you help plan or prepare meals/food?	3.3 ± 1.3
16. Do you keep home/room clean or clean-up after meals?	3.1 ± 1.3
**Total mean score**	3.4 ± 1.5

Legend: 1 - No, I do not know how. 2 - No, but I want to learn. 3 - No*,* but I am learning to do this. 4 - Yes, I have started doing this. 5 - Yes, I always do this when I need to

The mean value of the TRAQ questionnaires’ scores collected is 3.4±1.5 out of 5, and the mean score of SIBDQ is 53.9±9.8 out of 70.

**Fig. 3:**
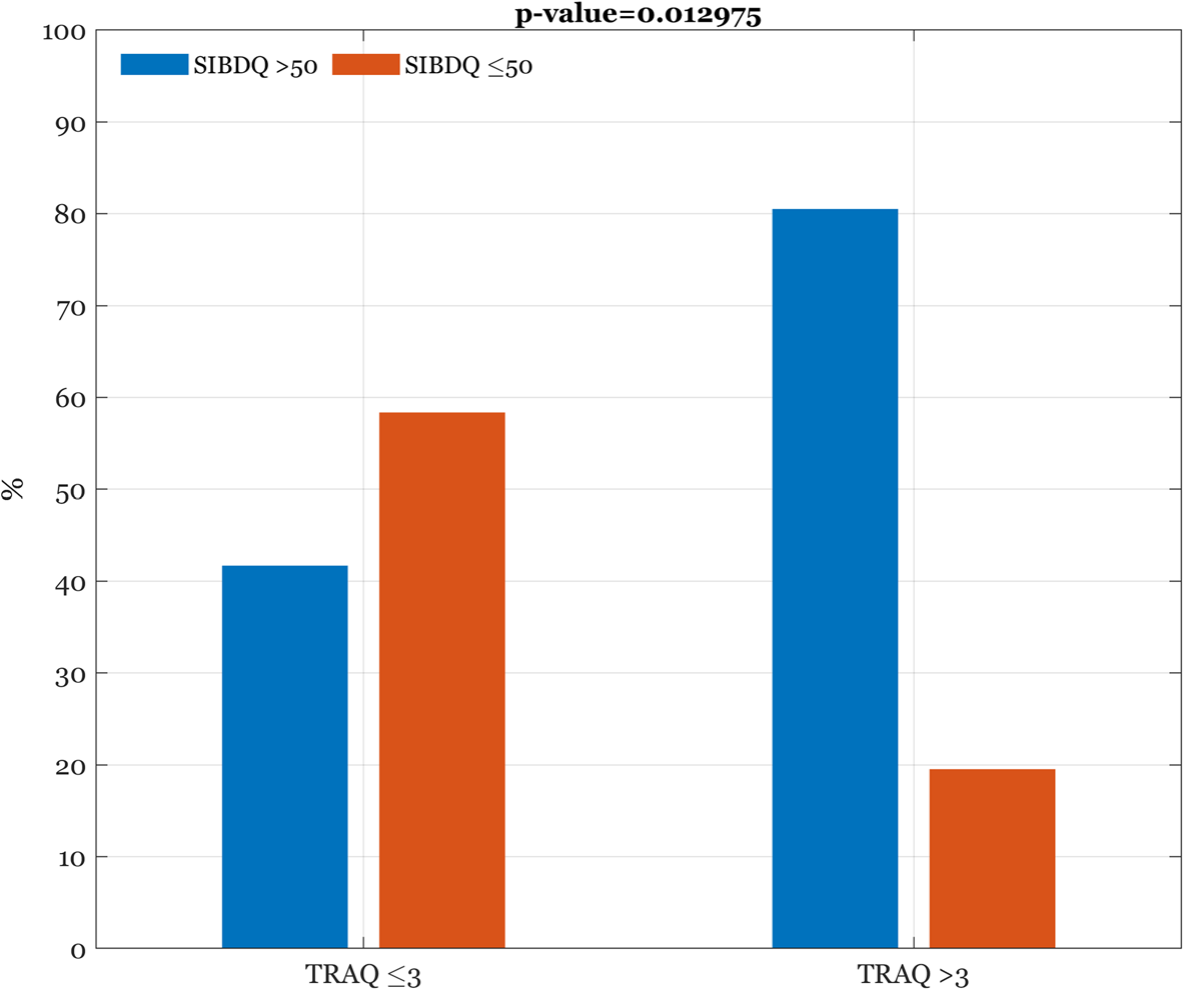
Association between TRAQ and SIBDQ scores

Considering as poor transition readiness a TRAQ score ≤ 3 out of 5, and as a good HRQoL a SIBDQ score > 50, significant association was found between a TRAQ mean score > 3 and a SIBDQ > 50 (*p*=0.0129). In fact, the 80.5% of patients with TRAQ mean score > 3 reported scores > 50 of SIBDQ, compared to the 41.7% of those with TRAQ mean score ≤ 3 (Fig. 3).

**Fig. 4:**
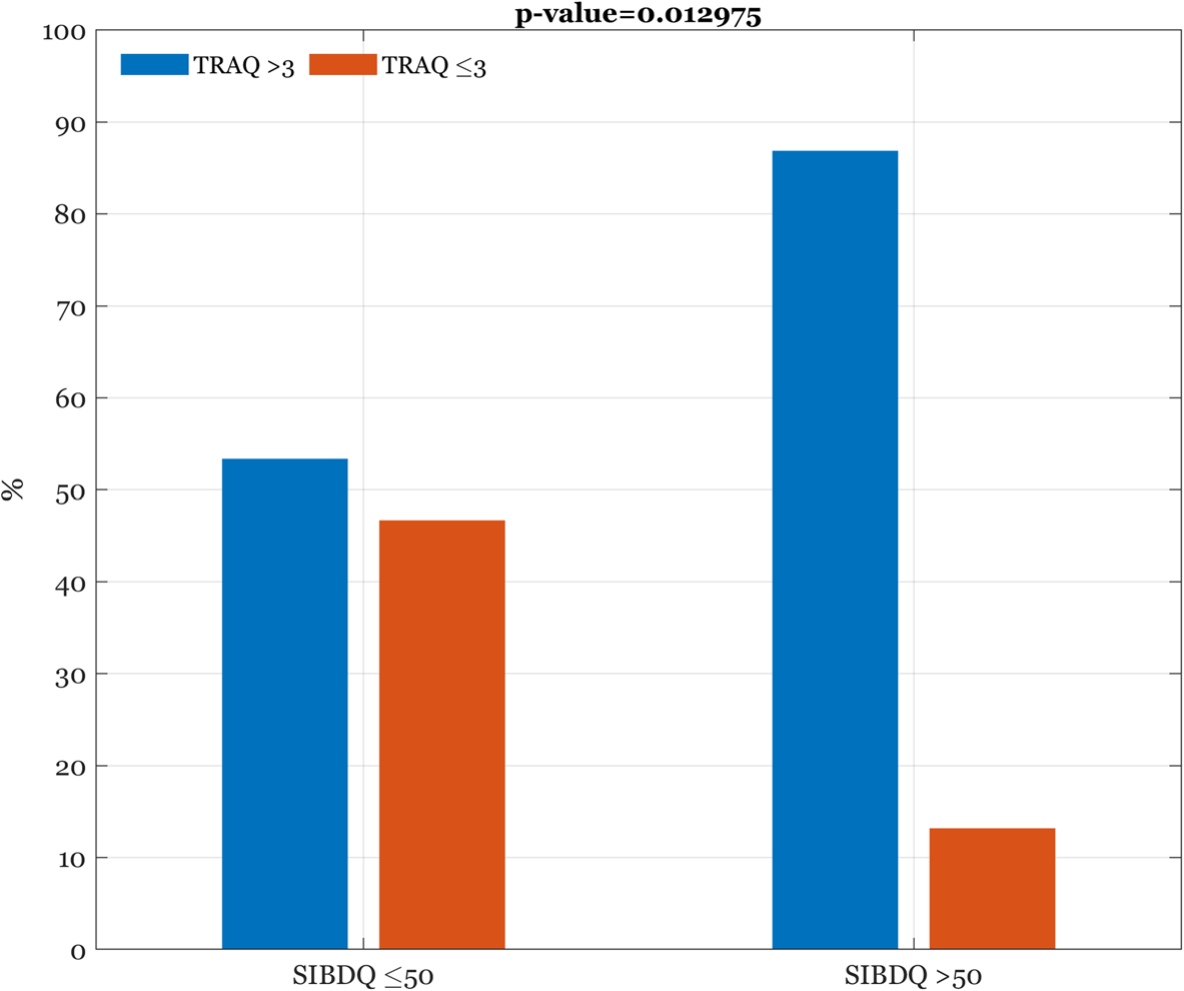
Association between SIBDQ and TRAQ scores

Conversely, considering patients referring a good HRQoL (SIBDQ > 50), the 86.8% reported TRAQ scores > 3, against the 13.2% that reported scores ≤ 3(Fig. 4).

No significant differences were observed among TRAQ mean scores of patients who completed transition within the age of 19 (3.3±0.5) compared to those who passed after 20 years (3.4±0.5), with *p*=0.36.

Overall, 75% of patients have a positive opinion of the transition model adopted. According to the answers collected, the three most important aspects for an ideal transition for patients are: timing of transition in a phase of stable remission (52% of patients), the access to scheduled joint visits with both pediatricians and adult gastroenterologists (49%) and the presence of psychologist and nutritionist figures in the adult center (34%). Less important, according to the patients’ opinion: the short distance between the two centers (24%), the possibility to choose independently the adult gastroenterologist (9%) and the protraction of transition for longer than 1 year (8%).

Forty percent of patients believe that the disease has delayed their educational and professional pathway, and 90% of patients who underwent surgery believe that surgery has overall improved their quality of life. The 88% of patients say they feel better now than they did in pediatric age, and 70% report an actual clinical well-being. The 45% of patients finally reported the presence of other cases of IBD in the family.

No significant difference (*p*> 0.05) has been found between patients coming from the two different centers in terms of questionnaire’s answers or short and long-term outcomes during follow-up visits.

## Discussion

We have explored the outcome of the transition process of 82 consecutive IBD patients coming from two pediatric referral hospitals to one IBD adult university hospital center, their readiness and the consequent impact on quality of life. Our model included two combined pediatric–adult visits, the first one at the children’s hospitals, in presence of parents, and the second one at the adult center. Despite there is no evidence that this model of transitional care is superior over others, the "joint-visit" model has been shown to be associated with a higher effectiveness on transmitting clinical data and building confidence in adults’ clinicians [14].

A recent study has been conducted on the transition process from pediatric to adult care of 106 young patients affected by IBD, investigating the 1-year success outcomes of this process [15]. This study has shown a significant maintenance or even a reduction of the disease activity scores and number of exacerbations in those patients who were in a remission phase at the time of transition. Our results confirm this data, strengthening the importance of the right timing of transition to the adult center.

For this reason, no strict chronological criteria have been considered in order to plan the timing of transition, and a case-by-case selection, according to IBD pediatricians’ judgment, has been used when possible.

The mean age observed at transition in our cohort was 20.1±2.7 years, exceeding the recommended ideal interval of the age of 17 and 19 [6]. In addition to this, patients who carried out transition with a moderate to severe disease activity have been resulted to be younger than those who transited with a mild or absent one (average age of 19.3±2.4 years vs. 20.2±2.5 years). This fact could be attributed to a lower engagement towards transition process made by patients and caregivers in those cases that did not present symptoms.

For this reason it can be also assumed that patients who transit on a younger age, and who often reach the adult hospital in need for hospitalization and surgery, making the experience of the transition even more traumatic, could be considered at higher risk of a further clinical deterioration in the first years of adulthood, and should need greater clinical care in order to obtain the best possible outcome in terms of quality of life, fundamental parameter to guarantee a good compliance to therapy in the future.

Data collected also show that main risk factors linked to a higher rate of surgery within the first year from transition can be considered the CD phenotype (particularly B2 and B3 behavior phenotypes), the number of biologics drugs assumed during the pediatric age, the clinical activity at diagnosis and the absence of an endoscopic or clinical remission.

An active disease at the time of transition has been already associated with an unsuccessful transition [16]. According to this, patients should complete transition to the adult center while in clinical remission or mild disease activity. Conversely, the transition of active patients should be rather delayed until the resolution of the acute phase, in order to avoid psychological traumatisms and to promote a greater confidence in the adult specialist and a good compliance with the therapy management [17].

Furthermore, it has been recently observed in an US cohort the absence of association between TRAQ scores and different measures of health [18]. In our cohort, instead, we found a significative positive association between TRAQ and SIBDQ questionnaires, highlighting the importance to assess a real readiness and demonstrating the association between patients’ transition readiness and higher HRQoL.

This fact gets even more important considering that we did not observe significant differences in terms of TRAQ mean scores among patients of different age, ant that we found more or less the same level of independence and awareness of disease within different groups, proving that transitions carried out at older ages do not improve readiness and quality of life score in significant way.

Our patients’ cohort expressed generally satisfaction about the transition process adopted, in particular about the opportunity to join combined pediatric-adult visits. Parents seem to still play a central role in patients’ lives and in their relationship with disease, especially for managing scheduled appointments, medications (including also keeping in memory of previous side effects or intolerance) and talking with providers.

This confirms the urgent need to structure educational programs during childhood, including also parents, in order to stimulate the acquisition by patients of a greater independence and capability of self-management.

Our study has some limitations, of which main ones can be considered the inclusion of two pediatric centers, with possible different therapy approaches, the single model of transition analyzed and the low percentage of children in remission enrolled between patients who carried out transition within the recommended age of 19 years.

The first limitation could lead to some biases related to the lack of homogeneity in the management and follow up, even no significant differences have been found in terms of short and long-term outcomes. Patients enrolled were similar in terms of age, and the same associations have been found in those who carried out transition in an active phase of disease, even if coming from different centers. On the other hand, our study could be considered a realistic model where patients with different backgrounds can converge to a single adult center through a standardized path.

In conclusion, our data support that mild activity of disease at transition and a high level of adolescent’s autonomy skills are the most important aspects of considering for transition and for assuring a better HRQoL in this difficult phase of care.

## Data Availability

The datasets used and analyzed during the current study are available from the corresponding author on reasonable request.
